# Malignant Anomalous Aortic Origin of the Left Main Coronary Artery Managed by Ostial Reimplantation: A Case Report

**DOI:** 10.1002/ccr3.71020

**Published:** 2025-10-27

**Authors:** Biruk T. Mengistie, Chernet T. Mengistie, Asteway M. Haile, Nahom D. Gerer, Samuel Mesfin Girma, Elezer B. Zewde

**Affiliations:** ^1^ School of Medicine, College of Health Sciences Addis Ababa University Addis Ababa Ethiopia

**Keywords:** anomalous aortic origin of a coronary artery, coronary computed tomography angiography (CCTA), coronary reimplantation, left main coronary artery anomaly

## Abstract

Anomalous origin of the left main coronary artery (LMCA) from the right coronary sinus with an interarterial course is a rare congenital variant associated with a high risk of myocardial ischemia and sudden cardiac death. We describe a 39‐year‐old man presenting with exertional chest pressure and dyspnea whose coronary CT angiography revealed an anomalous LMCA arising from the right sinus of Valsalva and coursing between the aorta and pulmonary artery without evidence of atherosclerosis. Transthoracic echocardiography was normal, and stress testing showed no inducible ischemia. The patient underwent surgical reimplantation of the anomalous LMCA into the left aortic sinus under cardiopulmonary bypass, with the right sinus defect closed using autologous pericardium. Recovery was uneventful, and postoperative imaging confirmed a widely patent LMCA. At three months, he remained asymptomatic with preserved ventricular function and no inducible ischemia. This case underscores the importance of high‐resolution imaging for diagnosis and demonstrates that reimplantation offers a safe and effective surgical option for malignant coronary anomalies lacking an intramural segment.

**Summary**

- Anomalous origin of the left main coronary artery from the right sinus with an interarterial course is a rare but potentially life‐threatening anomaly associated with sudden cardiac death in young adults.
- Coronary computed tomography angiography (CCTA) enables accurate diagnosis and anatomical delineation.
- When an intramural segment is absent, surgical reimplantation into the appropriate sinus is a safe and durable alternative to unroofing.
- Early recognition and timely surgical correction are crucial for favorable outcomes.

## 1 Introduction

Congenital coronary artery anomalies are uncommon, affecting approximately 0.2%–1.2% of the population [1]. One subset, anomalous aortic origin of a coronary artery (AAOCA), is categorized when a coronary artery arises from the opposite aortic sinus. Within AAOCA, an anomalous left main coronary artery (LMCA) arising from the right aortic sinus with an interarterial course is exceedingly rare (estimated prevalence ~0.02%–0.05%) [2, 3]. Despite its rarity, this variant carries a *malignant* phenotype. It is recognized as the highest‐risk coronary anomaly due to susceptibility to dynamic obstruction during exertion [2, 4]. Indeed, AAOCA is the second leading cause of sudden cardiac death in young athletes after hypertrophic cardiomyopathy [4, 5], and LMCA from the right sinus portends a higher risk of adverse events than anomalous right coronary artery origins [1].

Patients with malignant AAOCA are often asymptomatic or present with nonspecific exertional symptoms (chest pain, dyspnea, syncope) that reflect transient myocardial ischemia [3, 6]. The proposed mechanisms include an acute‐angle, slit‐like coronary ostium that limits flow, an intramural aortic wall segment that may spasm or occlude, and external compression of the interarterial course between the great vessels [2, 3, 7]. These dynamic factors can provoke ischemia during exercise and trigger arrhythmias or sudden death [2, 7].

Diagnosis of anomalous coronary origin and course is established by advanced imaging, most commonly coronary computed tomography angiography (CCTA), which precisely defines the origin and proximal path of the artery [2, 3]. Noninvasive tests (echocardiography, stress imaging) may be inconclusive if ischemia is intermittent [3, 8]. Once identified, current expert consensus recommends surgical correction of the malignant variant even in asymptomatic patients [9]. Surgical options include unroofing of an intramural segment, reimplantation of the coronary ostium into the correct sinus, or bypass grafting when other approaches are not feasible [4, 9, 10].

In this case, we report a middle‐aged man with exertional chest pain found to have an interarterial LMCA from the right sinus, who underwent successful coronary reimplantation. We highlight this management in the context of reported outcomes for similar anomalies. By reporting this case, we aim to increase clinical awareness, demonstrate the role of advanced imaging in detection, and contribute to the limited data guiding surgical decision‐making. While most published series focus on pediatric or adolescent patients, data on adult presentations and outcomes remain limited. Reporting adult cases is important because symptoms may be subtle, routine stress tests can be non‐diagnostic, and surgical decision‐making must balance risk and benefit. This case contributes to the literature by showcasing the role of high‐resolution imaging in diagnosis, the limitations of functional testing, and the safety of ostial reimplantation as an effective surgical option in an adult patient without an intramural segment.

## 2 Clinical History/Examination

A 39‐year‐old male presented with a three‐month history of exertional chest pain and shortness of breath. The symptoms were most pronounced during moderate physical activity and relieved with rest, suggesting an ischemic pattern. He reported no history of syncope, palpitations, or prior cardiovascular events. Past medical history was unremarkable, with no known hypertension, diabetes, or dyslipidemia, and there was no family history of premature coronary artery disease or sudden cardiac death. Physical examination revealed a well‐nourished man in no acute distress, with stable vital signs. Cardiovascular examination showed normal heart sounds without murmurs, gallops, or rubs. No signs of heart failure, such as peripheral edema or elevated jugular venous pressure, were present.

The patient denied palpitations, presyncope, or syncope. He reported mild exercise intolerance manifested by reduced tolerance for vigorous activity but stated that he was able to continue his usual occupational duties during the three months of symptoms. He works as an accountant. He had no prior history of congenital heart disease, no prior cardiac surgery or interventions, and no history of similar cardiac symptoms in childhood. There was no history suggestive of cyanosis during infancy or early childhood. Family history was negative for premature coronary artery disease or sudden cardiac death.

## 3 Differential Diagnosis, Investigations, and Treatment

**FIGURE 1:**
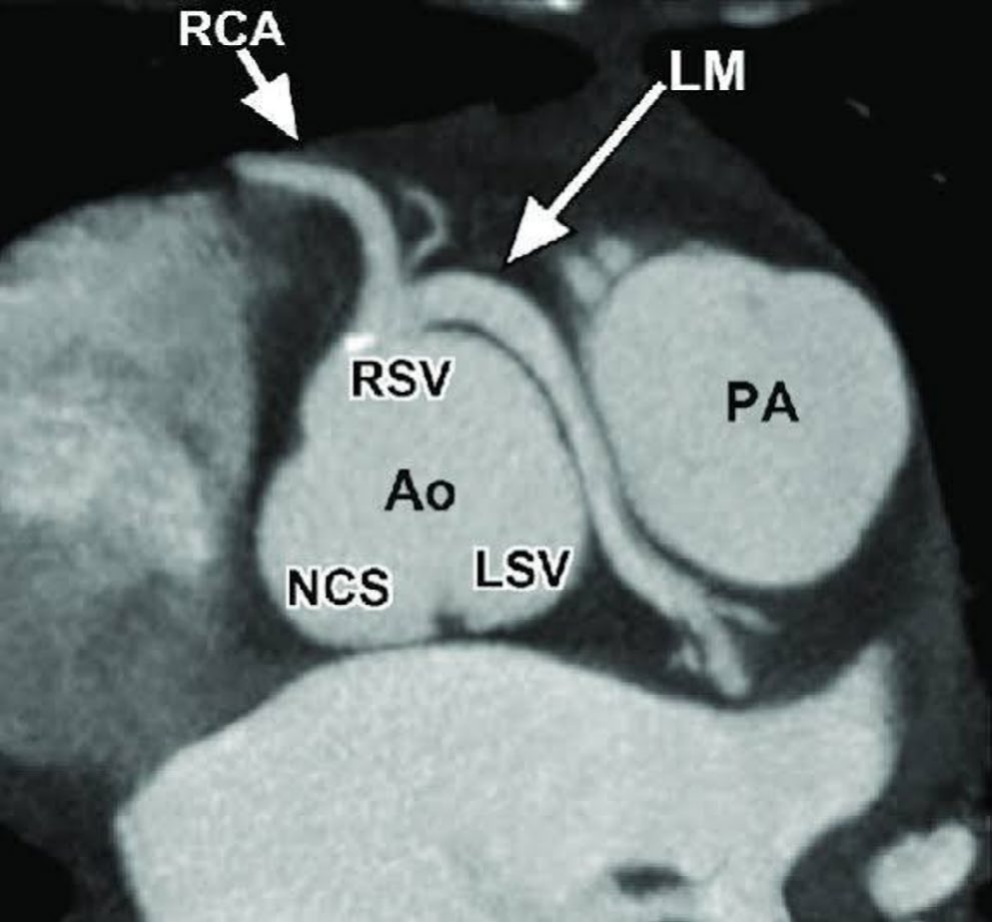
Coronary computed tomography angiogram showing the left main coronary artery (LM) arising anomalously from the right sinus of Valsalva (RSV, arrow), and coursing between the aorta (Ao) and pulmonary artery (PA). The right coronary artery (RCA) and left coronary sinus (LSV) are labeled.

**FIGURE 2:**
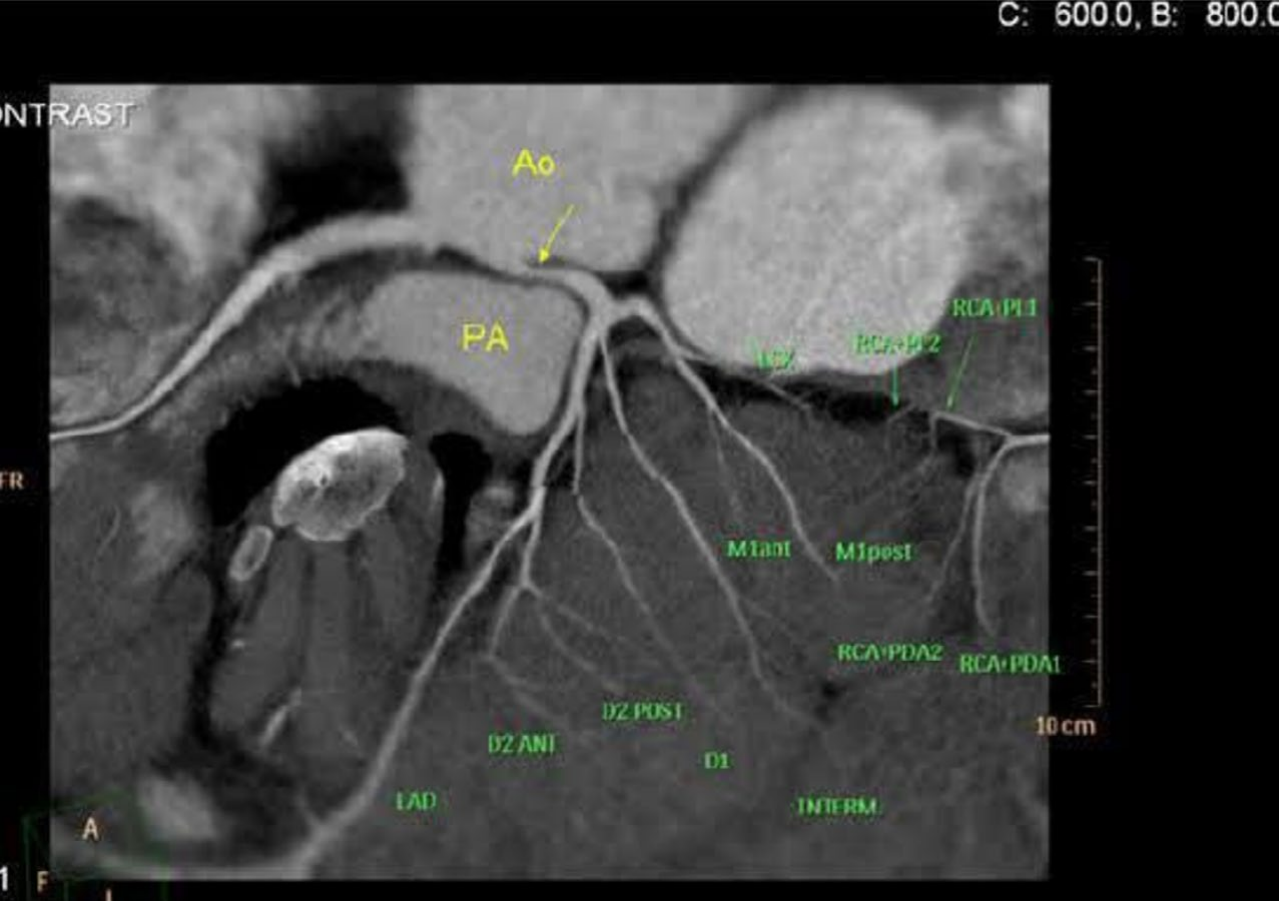
Multiplanar CCTA demonstrating the proximal left main (LM) artery originating from the right sinus (RSV) with an anterior course toward the left sinus. The aortic root (Ao) and pulmonary artery (PA) are visible.

Routine laboratory investigations were unremarkable. Baseline Electrocardiogram (ECG) and transthoracic echocardiography showed no ischemic changes and preserved biventricular function. Given his symptoms, CCTA was performed, which demonstrated the LMCA arising from the right coronary sinus through a separate ostium and coursing between the aorta and pulmonary artery (interarterial course) (Figures 1 and 2). No atherosclerotic coronary disease was identified. The patient underwent both nuclear myocardial perfusion imaging and stress echocardiography; neither study demonstrated inducible ischemia. In view of the malignant coronary anatomy, the patient was referred for surgical correction.

The patient underwent surgery under general anesthesia. Cardiopulmonary bypass (CPB) was established with bicaval cannulation after systemic heparinization, and myocardial protection was achieved with cold antegrade cardioplegia following systemic cooling to 32°C. The total CPB time was 193 min, with a cross‐clamp time of 99 min. Through a median sternotomy, the ascending aorta was cross‐clamped and transected above the sinotubular junction. The anomalous LMCA ostium was identified in the right sinus, excised as a button of aortic wall, and mobilized distally to its bifurcation into the left anterior descending coronary artery (LAD) and left circumflex coronary artery (LCx), sparing a branch to the sinoatrial node (SA). The left coronary sinus was prepared with an appropriately sized opening, and the LMCA button was reimplanted into the left sinus using fine polypropylene sutures. The defect in the right sinus was closed with an autologous pericardial patch, and the aorta was repaired. After meticulous de‐airing, the cross‐clamp was released, and the heart resumed sinus rhythm. The patient was successfully weaned from bypass without inotropic support; protamine was administered, and hemostasis was secured.

## 4 Outcome and Follow‐Up

The postoperative course was uneventful. The patient was extubated on the day of surgery, required only minimal Intensive Care Unit (ICU) support, and had no arrhythmias or ischemic changes. Serial echocardiograms and postoperative CCTA demonstrated normal ventricular function and widely patent coronaries. At 3‐month follow‐up, he remained asymptomatic, with normal exercise tolerance and no inducible ischemia on stress testing.

## 5 Discussion

Anomalous LMCA arising from the right sinus of Valsalva with an interarterial course is an exceptionally rare congenital variant. Coronary artery anomalies overall occur in roughly 0.2%–1% of individuals, and anomalous origin of the left main from the right sinus is reported in only ~0.02%–0.05% [1, 2, 3]. In contrast, anomalous right coronary origin is several‐fold more common [1]. Because of this rarity, there is limited large‐scale data, but published series emphasize its relative infrequency and importance. For example, a multicenter pediatric cohort had only 20% of cases involving the left coronary, the remainder being right coronary anomalies [11]. In our patient, the CCTA clearly showed the LMCA arising from the right sinus and passing between the aorta and pulmonary trunk, a high‐risk “interarterial” course.

Although malignant LMCA anomalies typically present in adolescence or young adulthood, some patients remain asymptomatic until middle age. Mechanisms proposed for delayed presentation include variability in ostial morphology, length of intramural segment, degree of dynamic compression, and differences in physical activity demands [2, 7]. Several adult case reports have described similar patients presenting in their 30s or 40s with exertional chest pain or incidental findings [3, 7, 8]. Our patient's late presentation underscores the importance of considering coronary anomalies even beyond the young athletic population.

Diagnostically, multimodality imaging is critical. Transthoracic echocardiography often misses these anomalies, whereas CT or MRI delineates the three‐dimensional course [2, 3, 8]. In this case, the absence of atherosclerosis and a “slit‐like” ostium to the LMCA were revealed by CCTA. Notably, ischemia may not be demonstrated on routine stress tests; our patient's nuclear and echocardiographic stress imaging were normal, despite his anomalous anatomy. This is concordant with reports that only specialized stress testing reliably detects transient ischemia in AAOCA [12]. The key imaging features distinguishing malignant AAOCA include an acute take‐off angle of the coronary ostium, an intramural aortic segment, and compression of the interarterial portion between the great vessels [3, 13]. Our patient's anomaly exhibited these hallmarks, explaining his exertional symptoms.

Routine stress tests can be normal in malignant AAOCA because ischemia is often dynamic and exertion‐dependent; transient compression of an interarterial segment, an acute‐angle/slit‐like ostium, or intermittent intramural narrowing may occur only under specific hemodynamic conditions, so a single test can miss ischemia [8, 9, 13]. Therefore, high‐resolution anatomic imaging (CCTA/MRI) and targeted functional assessments (stress perfusion MRI, CT‐FFR, or invasive physiologic testing) are important when clinical and imaging findings conflict [8, 10, 12]. Management should be individualized: symptomatic patients with malignant anatomy are generally recommended for surgical correction, while asymptomatic patients with high‐risk anatomic features are often offered surgery after Heart‐Team review; low‐risk variants may be managed conservatively with activity modification and surveillance [10, 14, 15].

Management of interarterial AAOCA is surgical. Unroofing of the intramural segment has been the most common approach for cases with a shared aortic wall, whereas translocation (reimplantation) or coronary ostioplasty are alternatives when no appreciable intramural course exists [4, 9, 10]. In our patient, the LMCA had a brief origin without significant intramural length, making direct reimplantation into the left sinus technically feasible. We elected this “ostial translocation” because it relocates the coronary into its native position and obviates extrinsic compression [9]. Bypass grafting of the anomalous vessel is reserved for situations where unroofing or translocation cannot be performed safely [9].

Published outcomes support the efficacy and safety of surgical repair for AAOCA. In a large pediatric series of 44 patients (median age 14), surgical correction (predominantly unroofing) was associated with no mortality, and 91% of patients were asymptomatic and 95% returned to full activity during follow‐up [11]. Likewise, a mixed pediatric‐adult cohort reported that both unroofing and reimplantation effectively relieved ischemia, with 94% of reimplantation patients and 93% of unroofing patients achieving unrestricted exercise postoperatively [14]. Another recent cohort of 230 patients undergoing AAOCA surgery (mostly pediatric) observed zero operative deaths and a very low rate of reintervention (2.6%) [15]. Notably, in that series, no patient in the reimplantation group required reoperation, whereas a small fraction of unroofing cases did, underscoring reimplantation as a durable solution. These outcomes mirror our patient's course: the surgery was uncomplicated, postoperative imaging confirmed widely patent coronaries, and he remains asymptomatic with preserved ventricular function.

Our choice of surgical reimplantation aligns with expert experience. Recent guidelines and reviews note that reimplantation is indicated when there is a minimal intramural course and the anomalous ostium can be safely relocated [9, 10]. In select high‐volume centers, this has become the preferred technique for many AAOCA cases because it addresses the pathologic origin directly [15]. However, it requires meticulous technique to avoid tension or kinking [14]. In the present case, the ostial button was mobilized sufficiently and reimplanted without branches' compromise, achieving an excellent result. Continued surveillance is warranted given the theoretical risk of late ostial narrowing, but published follow‐up suggests sustained relief of ischemia after reimplantation [14, 15]. This case adds to adult series showing that, when an intramural segment is absent, coronary ostial reimplantation is a safe and durable alternative to unroofing, with low operative morbidity and excellent mid‐term patency rates [14, 15].

While our patient's outcome at three months was favorable, longer‐term follow‐up is necessary to monitor for late complications such as ostial narrowing or graft failure [10, 15]. Published series have emphasized the importance of ongoing surveillance, particularly with imaging and stress testing at intervals, to ensure durable patency of the reimplanted coronary artery [14]. Our patient remains under regular cardiology follow‐up with plans for repeat imaging at one year and beyond.

In summary, this patient's anomaly, an interarterial LMCA from the right sinus, exemplifies a rare but critical diagnosis. Diagnosis relies on advanced imaging to identify the malignant course. Current evidence strongly favors surgical correction of such anomalies regardless of symptoms, and our case adds to the literature that coronary reimplantation can be performed safely with excellent short‐term and likely long‐term outcomes [11, 14, 15].

## 6 Conclusion

Anomalous left main coronary artery from the right sinus of Valsalva with an interarterial course is a rare congenital variant that poses a high risk for myocardial ischemia and sudden death. High‐resolution imaging (e.g., CCTA) is essential for accurate diagnosis. For malignant courses, prompt surgical correction is indicated even in asymptomatic patients. In this case, surgical reimplantation of the LMCA into the correct aortic sinus resulted in complete symptom resolution and normal postoperative cardiac function. Clinicians should maintain a high index of suspicion for coronary anomalies in young patients with exertional symptoms and use appropriate imaging. Early recognition and referral for surgical management can markedly improve outcomes in this population.

## 7 Patient Perspective

Before the operation, I often had chest pressure and shortness of breath, especially when walking quickly or climbing stairs. I was worried about my heart. After the surgery, I recovered well and I can now do my daily activities without symptoms. I am grateful that the doctors found the problem early and corrected it.

## Author Contributions

**Biruk T. Mengistie:** conceptualization, data curation, resources, writing – original draft. **Chernet T. Mengistie:** resources, writing – original draft. **Asteway M. Haile:** writing – original draft, writing – review and editing. **Nahom D. Gerer:** data curation, software, visualization. **Samuel Mesfin Girma:** data curation, writing – review and editing. **Elezer B. Zewde:** supervision, writing – review and editing.

## Ethics Statement

IRB review and approval was waived for this case report.

## Consent

Written informed consent for publication of the clinical details and accompanying images was obtained directly from the patient. The signed consent form (in both English and Amharic) is held by the corresponding author and can be provided to the Editor on request.

## Conflicts of Interest

The authors declare no conflicts of interest.

## Data Availability

The data underlying the results presented in this work is available within the manuscript.
